# Synergy between Florfenicol and Aminoglycosides against Multidrug-Resistant *Escherichia coli* Isolates from Livestock

**DOI:** 10.3390/antibiotics9040185

**Published:** 2020-04-16

**Authors:** Shukho Kim, Jung Hwa Woo, So Hyun Jun, Dong Chan Moon, Suk-Kyung Lim, Je Chul Lee

**Affiliations:** 1 Department of Microbiology, School of Medicine, Kyungpook National University, Daegu 41944, Korea; shukhokim@knu.ac.kr (S.K.); dasomi999@naver.com (J.H.W.); kkk016@nate.com (S.H.J.); 2 Bacterial Disease Division, Animal and Plant Quarantine Agency, Gimcheon-si, Gyeongsangbuk-do 39660, Korea; ansehdcks@korea.kr (D.C.M.); imsk0049@korea.kr (S.-K.L.)

**Keywords:** antimicrobial agent, combination therapy, synergy, florfenicol, aminoglycosides

## Abstract

The increasing prevalence of antimicrobial resistance and the laborious development of novel antimicrobial agents have limited the options for effective antimicrobial therapy. The combination of previously used antimicrobial agents represents an alternative therapy for multidrug-resistant (MDR) pathogens. The objective of this study was to investigate the synergistic effect of a florfenicol (FFL)-based combination with other antimicrobial agents against MDR *Escherichia coli* isolates from livestock using checkerboard assays and murine infection models. The FFL/amikacin (AMK) and FFL/gentamicin (GEN) combinations showed synergy against 10/11 and 6/11 MDR *E. coli* isolates in vitro, respectively. The combination of FFL with aminoglycosides (AMK or GEN) exhibited a better synergistic effect against MDR *E. coli* isolates than the cephalothin (CEF)/GEN or FFL/CEF combinations. The combination of FFL with AMK or GEN could reduce the emergence of resistant mutants in vitro. The FFL/AMK combination showed a higher survival rate of mice infected with MDR *E. coli* isolates than FFL or AMK alone. In summary, the combination of FFL with aminoglycosides (AMK or GEN) is highly effective against MDR *E. coli* isolates both in vitro and in vivo. Our findings may contribute to the discovery of an effective combination regimen against MDR *E. coli* infections in veterinary medicine.

## 1. Introduction

The worldwide increase of antimicrobial resistance is a serious concern in both human and animal health [1]. The prevalence of multidrug-resistant (MDR) bacterial pathogens has limited the options for effective antimicrobial therapy, and development of new therapeutics active against MDR bacteria is urgently needed [2,3]. However, the development of new antimicrobial agents has not paralleled the prevalence of resistant bacteria and few novel antimicrobial agents, including derivatives from existing classes of antimicrobials, have been developed during the last decade [4,5,6]. In this context, the combination of previously used antimicrobial agents is an alternative therapeutic option, which can also reserve new antimicrobial agents. Combination antimicrobial therapy is used to provide a broader antibacterial spectrum, prevent the emergence of resistant bacteria, and minimize toxicity in hosts [7,8]. The synergy between β-lactams and aminoglycosides and between trimethoprim and sulfamethoxazole has been well defined [9,10]. In addition, many antimicrobial combinations are commercially available for treatment of MDR bacterial infections in veterinary medicine.

Florfenicol (FFL), a fluorinated synthetic analog of thiamphenicol, is a broad-spectrum antimicrobial agent commonly used in veterinary medicine and aquaculture [11,12]. It binds to the 50S ribosomal subunit and inhibits protein synthesis. Unlike chloramphenicol (CHL), FFL does not induce aplastic anemia in humans, and it is used to treat systemic bacterial infections and infectious diseases associated with respiratory pathogens in cattle, pigs, cats, dogs, and fish [13]. In Korea, the annual consumption of amphenicols in veterinary medicine gradually increased from 63.8 tons in 2010 to 99.6 tons in 2018, and the resistance rate to CHL among *Escherichia coli* isolates from diseased animals in 2018 ranged from 63.5% in poultry to 85.1% in pigs [14]. Moreover, resistance to CHL among *E. coli* isolates from healthy poultry and pigs was 44.2% and 76.7% during the same period, respectively. Aminoglycosides display bactericidal activity against most Gram-negative aerobic and facultative anaerobic bacteria. Aminoglycosides have been used extensively to treat bacterial infections in veterinary medicine due to its cost effectiveness and reliable activity against serious Gram-negative bacterial infections, but the spread of resistance, its toxicity, and relatively long withdrawal periods can limit its usage in veterinary medicine [15]. The annual consumption of aminoglycosides in veterinary medicine was 57.9 tons in 2018 in Korea, and the resistance rate to streptomycin (STR) among *E. coli* isolates from diseased animals in 2018 was 66.7% in poultry and 78.3% in pigs [14]. These results suggest that FFL or aminoglycosides cannot be used alone to treat *E. coli* infections in livestock. Aminoglycosides must penetrate into the cytosol of bacteria to exert their effect. The penetration of aminoglycosides into the cytosol of bacteria is enhanced by other antimicrobial agents, such as β-lactams. FFL acts as an antibacterial modulator to multiple classes of antimicrobial agents through the alteration of bacterial membrane permeability and the subsequent increase in intracellular concentrations of the antimicrobial agents used in the combination [16]. The combination of FFL with thiamphenicol showed a synergistic effect against swine *Actinobacillus pleuropneumoniae* and *Pasteurella multocida* both in vitro and in vivo [16,17]. The combination of FFL with oxytetracycline was also active against *Pseudomonas aeruginosa* isolates in vitro [16]. Based on these results, this study assessed the antimicrobial activity of FFL and other antimicrobial agents in combination against MDR *E. coli* isolates from livestock in vitro and in vivo.

## 2. Results and Discussion

### 2.1. Combination of Amphenicols and Aminoglycosides against MDR E. coli Isolates In Vitro

Eleven MDR *E. coli* isolates were tested for drug interactions between amphenicols and aminoglycosides or β-lactams using checkerboard assays (Table 1). The *E. coli* isolates that were previously recovered from healthy and diseased animals in Korea and showed low resistance rates to gentamicin (GEN) and amikacin (AMK) [14] were selected for a combined treatment with aminoglycosides and FFL. Resistance to STR, kanamycin (KAN), GEN, and AMK was observed in 11, 8, 5, and 4 isolates, respectively (Table 1). Initially, the synergism between the β-lactams and aminoglycosides using checkerboard assays was assessed, but only two isolates showed synergy in the cephalothin (CEF)/GEN combination (Table 2, Table S1). With the combination of amphenicols and aminoglycosides, 10 and 6 isolates showed synergy in the FFL/AMK (Table S2) and FFL/GEN (Table S3) combinations, respectively. Synergy was observed in the CHL/GEN (Table S4) and CHL/AMK (Table S5) combinations for 6 and 8 isolates, respectively. No isolates exhibited synergy in the FFL/CEF combination (Table S6). Next, to determine whether the FFL/GEN or FFL/AMK combination could reduce the emergence of resistant bacteria compared to a single antimicrobial agent, two *E. coli* isolates, EC10 susceptible to FFL and GEN (Table S7) and EC15 susceptible to FFL and AMK, were cultured on a Mueller–Hinton agar plate containing single or two antimicrobial agents, and the frequency of mutant colonies was assessed after 24 h. The *E. coli* EC10 isolate was from pig feces. The FFL/GEN combination reduced the emergence of mutant colonies slightly in the EC10 isolate (Table 3). However, no mutant colonies were observed in the EC15 isolate with the FFL/AMK combination. Our results suggest that the FFL/AMK combination is the most active against veterinary MDR *E. coli* isolates, and this antimicrobial combination can prevent the emergence of resistant bacteria in vitro.

### 2.2. Combination of FFL and Aminoglycosides against MDR E. coli Isolates In Vivo

**Figure 1:**
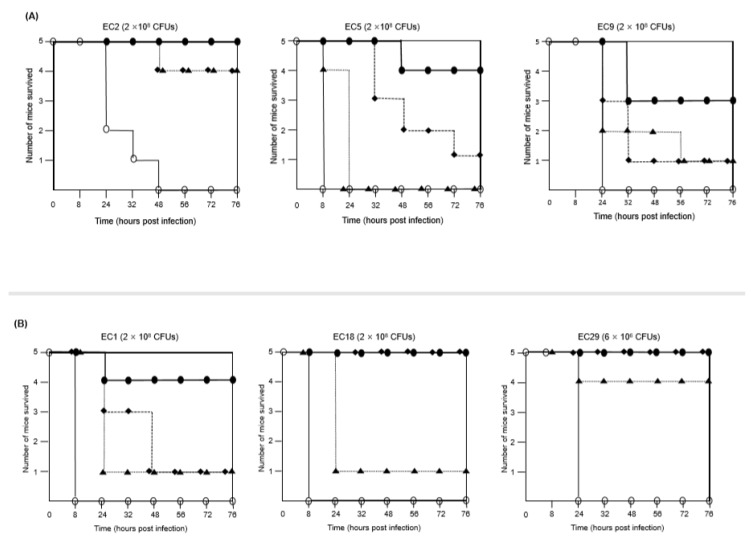
Treatment of mice infected with *E. coli* isolates. Mice were infected with *E. coli* isolates intraperitoneally. Bacterial inoculum size is indicated in parentheses. After 30 min, the mice received antimicrobial agents intramuscularly. (**A**) Mice infected with *E. coli* isolates (EC2, EC5, and EC9) were treated with PBS (○), FFL (▲, 20 mg/kg), AMK (◆, 10 mg/kg), or FFL (20 mg/kg)/AMK (●, 10 mg/kg). (**B**) Mice infected with *E. coli* isolates (EC1, EC18, and EC29) were treated with PBS (○), FFL (▲, 20 mg/kg), GEN (◆, 20 mg/kg), or FFL (20 mg/kg)/GEN (●, 20 mg/kg).

To determine whether the combination of FFL with aminoglycosides (AMK or GEN) was active against MDR *E. coli* isolates in vivo, mice were infected with *E. coli* intraperitoneally and then antimicrobials were injected intramuscularly. The three *E. coli* isolates in each group that showed synergy in the checkerboard assays, EC2, EC5, and EC9 for the combination of FFL/AMK and EC1, EC18, and EC29 for the combination of FFL/GEN, were selected for the combination therapy. Death was observed within 48 h in all the control mice treated with phosphate-buffered saline (PBS). The mice that were infected with either of the three *E. coli* isolates and then treated with the FFL/AMK combination exhibited a higher survival rate (60%–100%) than the infected animals treated with FFL (0%–80%) or AMK alone (20%–80%) (Figure 1A). The FFL/GEN combination showed the same survival rate (100%) of mice infected with two *E. coli* isolates, EC18 and EC29, compared to GEN alone (100%) (Figure 1B). Treatment of FFL alone reduced the survival rate (20%–80%) in mice infected with six *E. coli* isolates than with the combination of FFL/AMK (60%–100%) or FFL/GEN (80%–100%). These results suggest that the FFL/AMK combination is more effective in mice infected with the MDR *E. coli* isolates than FFL or AMK alone.

The emergence and spread of the *E. coli* isolates resistant to previously used antimicrobial agents, including veterinary, critically important antimicrobials, have forced veterinarians to use combinations of these antimicrobial agents. In vitro synergy tests can provide a guide for combination therapies, but it is important to determine the synergistic activity of these combinations in vivo. In Korea, amoxicillin/clavulanic acid (AMC) was highly active against *E. coli* isolates from diseased animals in 2018, with a resistant rate of 4.2%, whereas the same set of *E. coli* isolates was highly resistant to trimethoprim/sulfamethoxazole (53.5%) [14]. In this study, all isolates were resistant to amoxicillin (AMX) (MICs ≥ 512 µg/mL), but resistance to AMC was observed in one *E. coli* isolate. The current study assessed the synergy between β-lactams and aminoglycosides in combination against MDR *E. coli* isolates. The combination of CEF/GEN showed synergy in two isolates. It is necessary to develop new antimicrobial combination regimens for treatment of MDR *E. coli* infections in veterinary medicine. Based on the action mechanisms of FFL as an initial antibacterial modulator to multiple classes of antimicrobials [16], the current study assessed FFL-based combinations with aminoglycosides against MDR *E. coli* isolates. Checkerboard assays revealed that the combination of FFL with aminoglycosides was more active against MDR *E. coli* isolates than CEF/GEN or FFL/CEF in vitro. The FFL/AMK combination was the most active against MDR *E. coli* isolates and could prevent the emergence of resistant mutants. FFL was more active than CHL in the combination with AMK. The combination therapy with FFL/AMK or FFL/GEN was also promising against MDR *E. coli* isolates in vivo. Although the combination of FFL/GEN exhibited the same survival rates of mice compared to GEN alone in EC18 and EC29 isolates, the combination of FFL/GEN may reduce the emergence of resistant mutants. We tested representative MDR *E. coli* isolates from diseased livestock in Korea. It would be of interest to confirm the synergy between FFL and aminoglycosides by conducting studies with different clones or MDR *E. coli* strains originating from different geographical areas. The pharmacokinetics and pharmacodynamics of the FFL/AMK or FFL/GEN combinations should be also studied for clinical use, because the drug interactions between two antimicrobial agents and bacteria–host interactions cannot be mimicked utilizing in vitro models. In summary, the synergy between FFL and aminoglycosides against MDR *E. coli* isolates from livestock was demonstrated both in vitro and in vivo for the first time. Because MDR *E. coli* infections represent a major therapeutic challenge in veterinary medicine, our findings may contribute to the discovery of an effective combination regimen against veterinary MDR *E. coli* infections.

## 3. Materials and Methods

### 3.1. Bacterial Strains

A total of 12 MDR *E. coli* isolates were used in this study (Table 1 and Table S7). MDR *E. coli* isolates that were resistant to 5 or more different classes of antimicrobial agents were selected from a collection of *E. coli* isolates from fecal samples or tissue lesions of diseased animals at the diagnostic laboratory of the Bacterial Disease Division, Korea Animal and Plant Quarantine Agency, between 2011 and 2016. Twelve representative MDR *E. coli* isolates were then selected based on the antimicrobial resistance pattern, minimum inhibitory concentrations (MICs) of antimicrobial agents, carriage of aminoglycoside-modifying enzyme genes, geographical location of the farms, clinical samples, and source of animals. Eleven *E. coli* isolates were used to determine the synergism between the antimicrobial agents (Table 1). Two *E. coli* isolates, EC10 and EC15, were used to determine the mutation frequency in a combination of FFL and aminoglycosides. Nine and two isolates were from pigs and chicken, respectively. All *E. coli* isolates were obtained from the Korea Veterinary Culture Collection (KVCC). 

### 3.2. Antimicrobial Susceptibility Testing

The MICs of 13 antimicrobial agents were determined by the Sensititre susceptibility system using the KRNV4F Sensititre panel (Trek Diagnostic Systems) according to the manufacturer’s instructions. The MICs of AMK, KAN, and AMX were determined by the broth microdilution method according to the guidelines of the Clinical Laboratory Standards Institute (CLSI) [18]. *E. coli* American Type Culture Collection (ATCC) 25922 and *P. aeruginosa* ATCC 27853 were used as quality control strains. Interpretation of antimicrobial susceptibility was based on the guidelines of the CLSI when the MICs of the antimicrobial agents against the quality control strains were within the acceptable ranges. The breakpoints of ceftiofur, FFL, and STR were determined using the guidelines of the National Antimicrobial Resistance Monitoring System in Korea [19]. 

### 3.3. Polymerase Chain Reaction (PCR) Analysis of Aminoglycoside-Modifying Enzyme Genes

PCR was performed in a 20 µL volume containing 2 µL of boiled bacterial suspensions, 20 pM of each primer, 250 µM dNTPs, 10 mM Tris–HCl (pH 8.3), 50 mM KCl, 2.5 mM MgCl_2_, and 1.5 U of *Taq* DNA polymerase (Bioneer, Daejeon, Republic of Korea). Genes coding for aminoglycoside-modifying enzymes were amplified using primers specific for *aac(6’)-Ib, aac(3)-IIa, aac(3)-IVa, ant(2”)-Ia, ant(3”)-Ia, aph(3’)-Ia, aph(3”)-Ia*, and *aph(3”)-Ib* [20]. PCR conditions were followed as previously described [20].

### 3.4. Checkerboard Assay

In vitro synergy testing was performed using the checkerboard assay in a 96-well microplate. Preparation of bacteria and antimicrobial agents for the assay was conducted according to the CLSI guidelines [18]. In brief, bacteria (approximately 5 × 10^5^ colony forming units (CFUs)/mL) were prepared in cation-adjusted Mueller–Hinton broth (Difco) and inoculated in a 96-well microplate (100 μL/well). Two antimicrobial agents in each well were added and diluted two-fold, serially, horizontally and vertically. The fractional inhibitory concentration index (FICI) value was quantified using the following equation: FICI = FIC_A_ + FIC_B_ = (MIC of drug A in combination/MIC of drug A alone) + (MIC of drug B in combination/MIC of drug B alone). Drug interaction was classified as synergy, partial synergy, additivity, indifference, or antagonism [21,22]. Synergy, partial synergy, additivity, indifference, and antagonism were defined as FICI ≤ 0.5, 0.5 < FICI < 1, FICI = 1, 1 < FICI < 4, and FICI ≥ 4, respectively.

### 3.5. Mutation Frequency of E. coli Isolates

The mutation frequency was assessed from two *E. coli* isolates (EC10 and EC15) by exposing the bacteria to FFL, GEN, AMK, FFL/GEN, or FFL/AMK. *E. coli* EC10 and EC15 isolates were susceptible to FFL, GEN, and AMK. Bacteria (10^9^ CFUs) were spread onto Mueller–Hinton agar plates containing FFL (32 μg/mL), GEN (16 μg/mL), AMK (64 μg/mL), FFL (32 μg/mL)/GEN (16 μg/mL), and FFL (32 μg/mL)/AMK (64 μg/mL). The cultures were incubated at 37 °C overnight and then colonies were counted.

### 3.6. In Vivo Animal Experiments

Five-week-old female BALB/c mice (18–20 g) were purchased from Hyochang Science (Daegu, Korea) and housed under specific pathogen-free conditions. The animals were housed with five mice per cage in a laminar airflow room maintained at a temperature of 24 ± 2 °C, with a relative humidity of 55% ± 5% throughout the study. Mice were maintained for at least five days after caging and then six-week-old mice were used for experiments. The bacterial inoculum was prepared from an overnight culture of *E. coli* on blood agar plates and resuspended in PBS. The fifty percent lethal dose (LD_50_) was determined by inoculating groups of six mice intraperitoneally with serial 10-fold dilutions of *E. coli* isolates (https://www.aatbio.com/tools/ld50-calculator). The 5 × LD_50_ CFUs of bacteria (6 × 10^6^ CFUs/500 μL for EC29 and 2 × 10^8^ CFUs/500 μL for EC1, EC2, EC5, EC9, and EC18) were administered intraperitoneally. Thirty minutes after bacterial injection, 50 μL of FFL (20 mg/kg), GEN (20 mg/kg), or AMK (10 mg/kg) was injected intramuscularly into the right thigh for monotherapy, and 50 μL of FFL (20 mg/kg) and 50 μL of aminoglycosides (20 mg/kg for GEN or 10 mg/kg for AMK) were injected into the left and right thigh, respectively, for combination therapy [23,24,25,26,27]. Control animals received PBS injection in both thighs. Mortality was monitored for 76 h. All animal experiments were approved by Institutional Animal Care and Use Committee of Kyungpook National University (2018-0138).

## Figures and Tables

**Table 1:** Characteristics of the *E. coli* isolates used in this study.

Isolate No.	Animals	Samples	Isolated Year	Resistance Pattern	Aminoglycoside-Modifying Enzyme Gene
EC1	Pig	Feces	2016	STR, AMP, AMX, CEF, NAL, CIP, CHL, FFL, TET, SXT	*aph(3”)-Ia, aph(3”)-Ib*
EC2	Pig	Feces	2016	GEN, AMK, STR, KAN, AMP, AMX, NAL, CIP, TET, SXT	*aac(3)-IVa, ant(2”)-Ia, ant(3”)-Ia, aph(3’)-Ia*
EC5	Pig	Intestinal lesion	2015	COL, GEN, STR, KAN, AMP, AMX, TET, SXT	*aac(3)-IVa, ant(3”)-Ia, aph(3’)-Ia, aph(3”)-Ia, aph(3”)-Ib*
EC9	Pig	Feces	2016	STR, KAN, AMP, AMX, NAL, CIP, CHL, FFL, TET	*aph(3’)-Ia, aph(3”)-Ia, aph(3”)-Ib*
EC14	Chicken	Liver	2016	STR, AMP, AMX, TET, SXT	*ant(3”)-Ia*
EC15	Chicken	Oviduct	2016	STR, KAN, AMP, AMX, AMC, CEF, NAL, CIP, CHL, TET, SXT	*ant(3”)-Ia, aph(3’)-Ia, aph(3”)-Ia, aph(3”)-Ib*
EC18	Pig	Feces	2016	AMK, STR, KAN, AMP, AMX, CEF, NAL, CIP, CHL, FFL, TET, SXT	*aac(6’)-Ib, ant(2”)-Ia, ant(3”)-Ia, aph(3’)-Ia, aph(3”)-Ia, aph(3”)-Ib*
EC19	Pig	Intestinal lesion	2016	GEN, STR, AMP, AMX, CHL, FFL, TET, SXT	*aac(3)-IIa, ant(2”)-Ia, ant(3”)-Ia, aph(3”)-Ia, aph(3”)-Ib*
EC24	Pig	Urinary tract	2015	AMK, STR, KAN, AMP, AMX, CEF, CHL, FFL, TET, SXT	*ant(2”)-Ia, ant(3”)-Ia, aph(3’)-Ia, aph(3”)-Ia, aph(3”)-Ib*
EC28	Pig	Intestinal lesion	2015	COL, GEN, AMK, STR, KAN, AMP, AMX, CHL, FFL, TET	*ant(2”)-Ia, ant(3”)-Ia, aph(3”)-Ia, aph(3”)-Ib*
EC29	Pig	Feces	2011	COL, GEN, STR, KAN, AMP, AMX, NAL, CIP, CHL, FFL, TET, SXT	*aac(3)-IVa, ant(2”)-Ia, ant(3”)-Ia, aph(3’)-Ia, aph(3”)-Ia, aph(3”)-Ib*

Abbreviations: COL, colistin; GEN, gentamicin; AMK, amikacin; STR, streptomycin; KAN, kanamycin; AMP, ampicillin; AMX, amoxicillin; AMC, amoxicillin/clavulanic acid; CEF, cephalothin; XNL, ceftiofur; NAL, nalidixic acid; CIP, ciprofloxacin; CHL, chloramphenicol; FFL, florfenicol; TET, tetracycline; SXT, trimethoprim/sulfamethoxazole (1:19).

**Table 2:** In vitro inhibitory activity of different antimicrobial combinations against multidrug-resistant *E. coli* isolates.

Antimicrobial Combination	Number of Isolates (%)			
	Synergy (FICI ≤ 0.5)	Partial Synergy (0.5 < FICI < 1)	Additive (FICI = 1)	Indifference (1 < FICI < 4)
CEF/GEN	2 (18.2)	2 (18.2)	0	7 (63.6)
FFL/GEN	6 (54.5)	3 (27.3)	0	2 (18.2)
FFL/AMK	10 (90.9)	1 (9.1)	0	0
CHL/GEN	6 (54.5)	3 (27.3)	0	2 (18.2)
CHL/AMK	8 (72.7)	3 (27.3)	0	0
FFL/CEF	0	3 (27.3)	3 (27.3)	5 (45.5)

Abbreviations: FICI, fractional inhibitory concentration index; CEF, cephalothin; GEN, gentamicin; FFL, florfenicol; AMK, amikacin; CHL, chloramphenicol.

**Table 3:** Mutation frequency of *E. coli* isolates with the florfenicol and aminoglycosides combination.

*E. coli* Isolates	Frequency of Mutants Growing in Media Containing Antimicrobial Agents		
EC10 (MIC of FFL, 4 μg/mL; MIC of GEN, 4 μg/mL)	FFL (32 μg/mL)	GEN (16 μg/mL)	FFL (32 μg/mL) and GEN (16 μg/mL)
	2.3 × 10^−5^	5.2 × 10^−5^	8 × 10^−6^
EC15 (MIC of FFL, 4 μg/mL; MIC of AMK, 8 μg/mL)	FFL (32 μg/mL)	AMK (64 μg/mL)	FFL (32 μg/mL) and AMK (64 μg/mL)
	2 × 10^−7^	1.4 × 10^−6^	0

## Supplementary Materials

The following are available online at https://www.mdpi.com/2079-6382/9/4/185/s1, Table S1: In vitro synergy between cephalothin and gentamicin in combination against 11 multidrug-resistant *E. coli* isolates. Table S2: In vitro synergy between florfenicol and amikacin in combination against 11 multidrug-resistant *E. coli* isolates. Table S3: In vitro synergy between florfenicol and gentamicin in combination against 11 multidrug-resistant *E. coli* isolates. Table S4: In vitro synergy between chloramphenicol and gentamicin in combination against 11 multidrug-resistant *E. coli* isolates. Table S5: In vitro synergy between chloramphenicol and amikacin in combination against 11 multidrug-resistant *E. coli* isolates. Table S6: In vitro synergy between florfenicol and cephalothin in combination against 11 multidrug-resistant *E. coli* isolates. Table S7: Minimum inhibitory concentrations (MICs) of 16 antimicrobial agents against 12 *E. coli* isolates used in this study.
