# Supplementary material for: Synergy between Florfenicol and Aminoglycosides against Multidrug-Resistant Escherichia coli Isolates from Livestock
Source: Antibiotics (Basel). 2020 Apr 16;9(4):185. doi: 10.3390/antibiotics9040185 (PMC7235850; doi:10.3390/antibiotics9040185)
Supplement: Supplementary file 1 [file antibiotics-09-00185-s001.pdf]

antibiotics

MDPI

Table S1. In vitro synergy between cephalothin and gentamicin in combination against 11 multidrug-resistant *E. coli* isolates.

Isolate	MIC (μg/mL)		FIC		FICI	Drug Interaction[a]
No.	CEF	GEN	CEF	GEN		
EC1	128	8	0.002	1.0	1.002	Indifference
EC2	4	512	0.5	1.0	1.5	Indifference
EC5	8	32	0.125	1.0	1.125	Indifference
EC9	16	4	0.063	1.0	1.063	Indifference
EC14	8	4	0.125	1.0	1.125	Indifference
EC15	128	4	0.002	0.5	0.502	Partial synergy
EC18	128	4	0.002	1.0	1.002	Indifference
EC19	8	256	0.004	0.5	0.504	Partial synergy
EC24	64	4	0.002	1.0	1.002	Indifference
EC28	8	128	0.002	0.25	0.252	Synergy
EC29	4	64	0.004	0.25	0.254	Synergy

* Abbreviations: MIC, minimum inhibitory concentration; FIC, fractional inhibitory concentration; FICI, fractional inhibitory concentration index; CEF, cephalothin; GEN, gentamicin. [a] Drug interaction was classified into synergy, partial synergy, additivity, indifference, and antagonism that were FICI ≤ 0.5, 0.5 < FICI < 1, FICI = 1, 1 < FICI < 4, and FICI ≥ 4, respectively.

Table S2. In vitro synergy between florfenicol and amikacin in combination against 11 multidrug-resistant *E. coli* isolates.

Isolate	MIC (μg/mL)		FIC		FICI	Drug Interaction[a]
No.	FFL	AMK	FFL	AMK		
EC1	128	8	0.031	0.004	0.035	Synergy
EC2	4	512	0.25	0.002	0.252	Synergy
EC5	64	4	0.031	0.031	0.062	Synergy
EC9	128	8	0.031	0.004	0.035	Synergy
EC14	4	8	0.25	0.002	0.252	Synergy
EC15	4	16	0.5	0.063	0.563	Partial synergy
EC18	256	64	0.031	0.016	0.047	Synergy
EC19	256	8	0.031	0.25	0.281	Synergy
EC24	256	64	0.031	0.016	0.047	Synergy
EC28	128	512	0.031	0.002	0.032	Synergy
EC29	256	2	0.016	0.008	0.024	Synergy

* Abbreviations: MIC, minimum inhibitory concentration; FIC, fractional inhibitory concentration; FICI, fractional inhibitory concentration index; FFL, florfenicol; AMK, amikacin. [a] Drug interaction was classified into synergy, partial synergy, additivity, indifference, and antagonism that were FICI ≤ 0.5, 0.5 < FICI < 1, FICI = 1, 1 < FICI < 4, and FICI ≥ 4, respectively.

Table S3. In vitro synergy between florfenicol and gentamicin in combination against 11 multidrug-resistant *E. coli* isolates.

Isolate	MIC (μg/mL)		FIC		FICI	Drug Interac tion[a]
No.	FFL	GEN	FFL	GEN		
EC1	128	8	0.063	0.002	0.065	Synergy
EC2	8	512	1.000	0.008	1.008	Indifference
EC5	64	64	0.250	0.063	0.313	Synergy
EC9	128	4	0.016	0.500	0.516	Partial synergy
EC14	4	4	0.063	0.008	0.071	Synergy
EC15	4	4	1.000	0.004	1.004	Indifference
EC18	256	4	0.016	0.002	0.018	Synergy
EC19	256	512	0.500	0.016	0.516	Partial synergy

EC24	256	4	0.031	0.004	0.035	Synergy
EC28	64	128	0.500	0.008	0.508	Partial synergy
EC29	128	64	0.125	0.031	0.156	Synergy

* Abbreviations: MIC, minimum inhibitory concentration; FIC, fractional inhibitory concentration; FICI, fractional inhibitory concentration index; FFL, florfenicol; GEN, gentamicin. [a] Drug interaction was classified into synergy, partial synergy, additivity, indifference, and antagonism that were FICI ≤ 0.5, 0.5 < FICI < 1, FICI = 1, 1 < FICI < 4, and FICI ≥ 4, respectively.

Table S4. In vitro synergy between chloramphenicol and gentamicin in combination against 11 multidrug-resistant *E. coli* isolates.

Isolate	MIC (µg/mL)		FIC		FICI	Drug Interaction[a]
No.	CHL	GEN	CHL	GEN		
EC1	128	8	0.063	0.002	0.065	Synergy
EC2	16	512	1.0	0.002	1.002	Indifference
EC5	64	64	0.125	0.002	0.127	Synergy
EC9	128	4	0.063	0.002	0.065	Synergy
EC14	16	4	1.0	0.002	1.002	Indifference
EC15	32	4	0.25	0.50	0.75	Partial synergy
EC18	128	4	0.063	0.002	0.065	Synergy
EC19	128	512	0.063	0.25	0.313	Synergy
EC24	64	4	0.25	0.5	0.75	Partial synergy
EC28	64	128	0.25	0.016	0.266	Synergy
EC29	128	64	0.063	0.5	0.563	Partial synergy

* Abbreviations: MIC, minimum inhibitory concentration; FIC, fractional inhibitory concentration; FICI, fractional inhibitory concentration index; CHL, chlorqamphenicol; GEN, gentamicin. [a] Drug interaction was classified into synergy, partial synergy, additivity, indifference, and antagonism that were FICI ≤ 0.5, 0.5 < FICI < 1, FICI = 1, 1 < FICI < 4, and FICI ≥ 4, respectively.

Table S5. In vitro synergy between chloramphenicol and amikacin in combination against 11 multidrug-resistant *E. coli* isolates.

Isolate	MIC (µg/mL)		FIC		FICI	Drug Interaction[a]
No.	CHL	AMK	CHL	AMK		
EC1	128	8	0.25	0.25	0.5	Synergy
EC2	16	512	0.5	0.002	0.502	Partial synergy
EC5	64	4	0.063	0.016	0.079	Synergy
EC9	128	8	0.031	0.016	0.047	Synergy
EC14	8	8	0.5	0.016	0.516	Partial synergy
EC15	64	8	0.5	0.016	0.516	Partial synergy
EC18	128	64	0.063	0.016	0.079	Synergy
EC19	128	8	0.031	0.25	0.281	Synergy
EC24	64	64	0.016	0.25	0.266	Synergy
EC28	128	512	0.008	0.016	0.024	Synergy
EC29	128	2	0.063	0.016	0.079	Synergy

* Abbreviations: MIC, minimum inhibitory concentration; FIC, fractional inhibitory concentration; FICI, fractional inhibitory concentration index; CHL, chloramphenicol; AMK, amikacin. [a] Drug interaction was classified into synergy, partial synergy, additivity, indifference, and antagonism that were FICI ≤ 0.5, 0.5 < FICI < 1, FICI = 1, 1 < FICI < 4, and FICI ≥ 4, respectively.

Table S6. In vitro synergy between florfenicol and cephalothin in combination against 11 multidrug-resistant *E. coli* isolates.

Isolate	MIC (µg/mL)		FIC		FICI	Drug Interaction[a]
No.	FFL	CEF	FFL	CEF		
EC1	128	256	0.5	0.25	0.75	Partial synergy
EC2	8	4	0.25	1.0	1.25	Indifference
EC5	64	8	0.5	0.125	0.625	Partial synergy

EC9	128	16	0.5	0.5	1.0	Additive
EC14	4	8	0.5	1.0	1.5	Indifference
EC15	4	128	0.5	0.25	0.75	Partial synergy
EC18	128	128	0.5	0.5	1.0	Additive
EC19	128	8	0.5	1.0	1.5	Indifference
EC24	256	64	0.5	1.0	1.5	Indifference
EC28	128	8	0.5	0.5	1.0	Additive
EC29	256	4	0.5	1.0	1.5	Indifference

* Abbreviations: MIC, minimum inhibitory concentration; FIC, fractional inhibitory concentration; FICI, fractional inhibitory concentration index; FFL, florfenicol; CEF, cephalothin. [a] Drug interaction was classified into synergy, partial synergy, additivity, indifference, and antagonism that were FICI ≤ 0.5, 0.5 < FICI < 1, FICI = 1, 1 < FICI < 4, and FICI ≥ 4, respectively.

Table S7. Minimum inhibitory concentrations (MICs) of 16 antimicrobial agents against 12 *E. coli* isolates used in this study.

Isolate No.	MIC (μg/mL)															
	COL	GEN	AMK	STR	KAN	AMP	AMX	AMC	CEF	XNL	NAL	CIP	CHL	FFL	TET	SXT
EC1	≤2	8	8	>128	8	>64	>512	4	>64	>8	>128	16	>64	>64	64	>4/76
EC2	≤2	>64	>128	64	>128	>64	>512	8	4	≤0.5	>128	8	16	4	128	>4/76
EC5	32	32	4	128	>128	>64	>512	8	8	≤0.5	16	≤0.12	>64	64	64	≤0.12/2.28
EC9	≤2	4	8	128	>128	>64	>512	8	16	≤0.5	>128	>16	>64	>64	128	0.5/9.5
EC10	≤2	4	8	16	>128	4	>512	8	16	≤0.5	≤2	≤0.12	4	4	64	0.5/9.5
EC14	≤2	4	8	64	8	>64	>512	8	8	≤0.5	≤2	≤0.12	8	4	64	>4/76
EC15	≤2	2	8	>128	>128	>64	>512	64	>64	8	>128	8	32	4	128	>4/76
EC18	≤2	4	64	>128	>128	>64	512	16	>64	8	>128	>16	>64	>64	64	>4/76
EC19	≤2	>64	4	32	32	>64	>512	8	8	≤0.5	4	0.5	>64	>64	32	>4/76
EC24	8	4	64	>128	>128	>64	>512	8	32	≤0.5	16	0.5	>64	>64	128	>4/76
EC28	32	>64	>128	>128	>128	>64	>512	8	8	≤0.5	16	≤0.12	64	64	64	≤0.12/2.28
EC29	32	64	2	>128	>128	64	>512	4	4	0.5	128	16	>64	64	128	4/76

*Abbreviations: COL, colistin; GEN, gentamicin; AMK, amikacin; STR, streptomycin; KAN, kanamycin; AMP, ampicillin; AMX, amoxicillin; AMC, amoxicillin/clavulanic acid; CEF, cephalothin; XNL, ceftiofur; NAL, nalidixic acid; CIP, ciprofloxacin; CHL, chloramphenicol; FFL, florfenicol; TET, tetracycline; SXT, trimethoprim/sulfamethoxazole (1:19).
